# S100P is a molecular determinant of E-cadherin function in gastric cancer

**DOI:** 10.1186/s12964-019-0465-9

**Published:** 2019-11-25

**Authors:** Patrícia Carneiro, Ana Margarida Moreira, Joana Figueiredo, Rita Barros, Patrícia Oliveira, Maria Sofia Fernandes, Anabela Ferro, Raquel Almeida, Carla Oliveira, Fátima Carneiro, Fernando Schmitt, Joana Paredes, Sérgia Velho, Raquel Seruca

**Affiliations:** 1 Instituto de Investigação e Inovação em Saúde (i3S), Epithelial Interactions in Cancer Group, Rua Alfredo Allen 208, 4200-135 Porto, Portugal; 2 Institute of Molecular Pathology and Immunology of the University of Porto (IPATIMUP), Porto, Portugal; 3 Institute of Biomedical Sciences Abel Salazar, University of Porto (ICBAS), Porto, Portugal; 4 Department of Pathology and Oncology, Medical Faculty of the University of Porto, Porto, Portugal; 5 Department of Biology, Science Faculty of the University of Porto, Porto, Portugal; 6 Centro Hospitalar São João, Porto, Portugal

**Keywords:** Gastric cancer, S100P, E-cadherin, Prognosis, Survival

## Abstract

**Background:**

E-cadherin has been awarded a key role in the aetiology of both sporadic and hereditary forms of gastric cancer. In this study, we aimed to identify molecular interactors that influence the expression and function of E-cadherin associated to cancer.

**Methods:**

A data mining approach was used to predict stomach-specific candidate genes, uncovering S100P as a key candidate. The role of S100P was evaluated through in vitro functional assays and its expression was studied in a gastric cancer tissue microarray (TMA).

**Results:**

S100P was found to contribute to a cancer pathway dependent on the context of E-cadherin function. In particular, we demonstrated that S100P acts as an E-cadherin positive regulator in a wild-type E-cadherin context, and its inhibition results in decreased E-cadherin expression and function. In contrast, S100P is likely to be a pro-survival factor in gastric cancer cells with loss of functional E-cadherin, contributing to an oncogenic molecular program. Moreover, expression analysis in a gastric cancer TMA revealed that S100P expression impacts negatively among patients bearing Ecad^−^ tumours, despite not being significantly associated with overall survival on its own.

**Conclusions:**

We propose that S100P has a dual role in gastric cancer, acting as an oncogenic factor in the context of E-cadherin loss and as a tumour suppressor in a functional E-cadherin setting. The discovery of antagonist effects of S100P in different E-cadherin contexts will aid in the stratification of gastric cancer patients who may benefit from S100P-targeted therapies.

**Graphical abstract:**

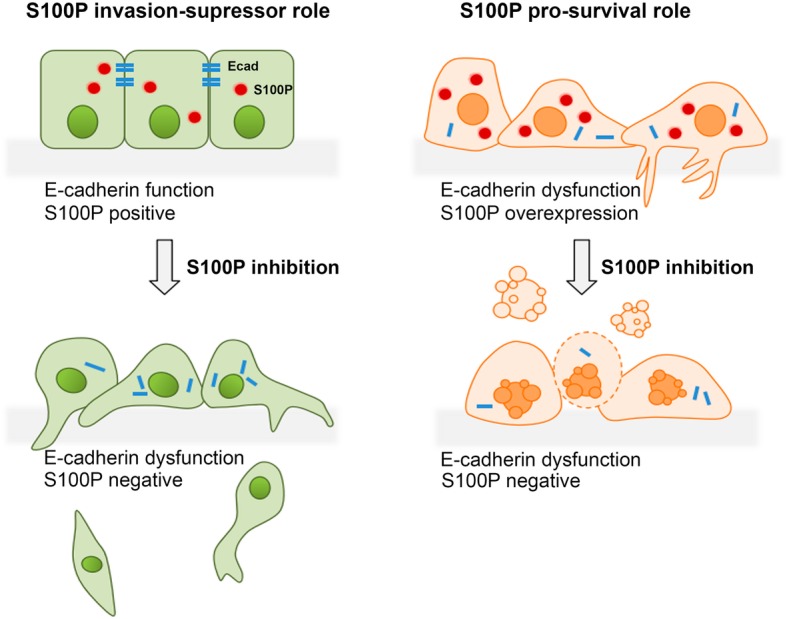

## Background

Gastric cancer (GC) remains a major clinical concern as one of the leading causes of cancer death worldwide, despite a steady decline in incidence in most developed countries [1, 2]. Notwithstanding significant progresses in surgical procedures and therapeutic regimens, the inherent molecular complexity and tumour heterogeneity of GC hamper the identification of specific biomarkers for early diagnosis [3, 4]. Further, the silent and asymptomatic nature of early stages of GC contribute to diagnosis at an advanced stage of disease and consequent poor patient prognosis [5].

Among the plethora of genetic mutations, epigenetic alterations and aberrant molecular signalling pathways known to be involved in GC development [6], E-cadherin has been awarded a key role in the aetiology of both sporadic and hereditary forms of the disease [7–9].

Throughout the past decades evidence has emerged demonstrating a network of signalling pathways that is able to intersect with the regulation and function of this adhesive molecule [5, 10]. However, knowledge is scarce regarding how specific molecular partners and mechanisms regulate E-cadherin expression and function in the stomach contributing to the pathophysiology of GC.

Herein, we report S100P as a putative E-cadherin regulator in the gastric epithelium. S100P is a member of the EF-hand calcium-binding family of S100 proteins that regulate a myriad of cellular processes in a Ca^2+^-dependent manner [11]. S100 proteins undergo multiple conformational changes in the presence of divalent calcium cations impacting on their affinity for interacting partners and, in fact, it has been suggested that S100 proteins are endowed with a great deal of flexibility and can coordinate multiple interactions with diverse target proteins [11, 12]. S100P has been reported to interact with a number of proteins both extracellularly and intracellularly, and its over expression in varied human cancers has been associated with disease progression, acquisition of chemoresistance and poor prognosis [12]. In fact, S100P is regarded as a potential drug target and the development of anti-S100P specific therapies has been considerably addressed, mainly in pancreatic cancer [13, 14].

Interestingly, in normal adult tissues, the highest levels of S100P are detected in placenta and in the stomach [15]. In this study, our data demonstrates that S100P has a dual role depending on the context of E-cadherin expression. In a wild-type E-cadherin context, S100P acts as an E-cadherin positive regulator, and its inhibition results in decreased E-cadherin expression and function, affecting cell invasive behavior. In contrast, in a dysfunctional E-cadherin setting, S100P is likely to be a molecular determinant allowing gastric cells to survive, thus contributing to an oncogenic pathway.

## Methods

### Definition of the list of putative stomach-specific genes

**Fig. 1:**
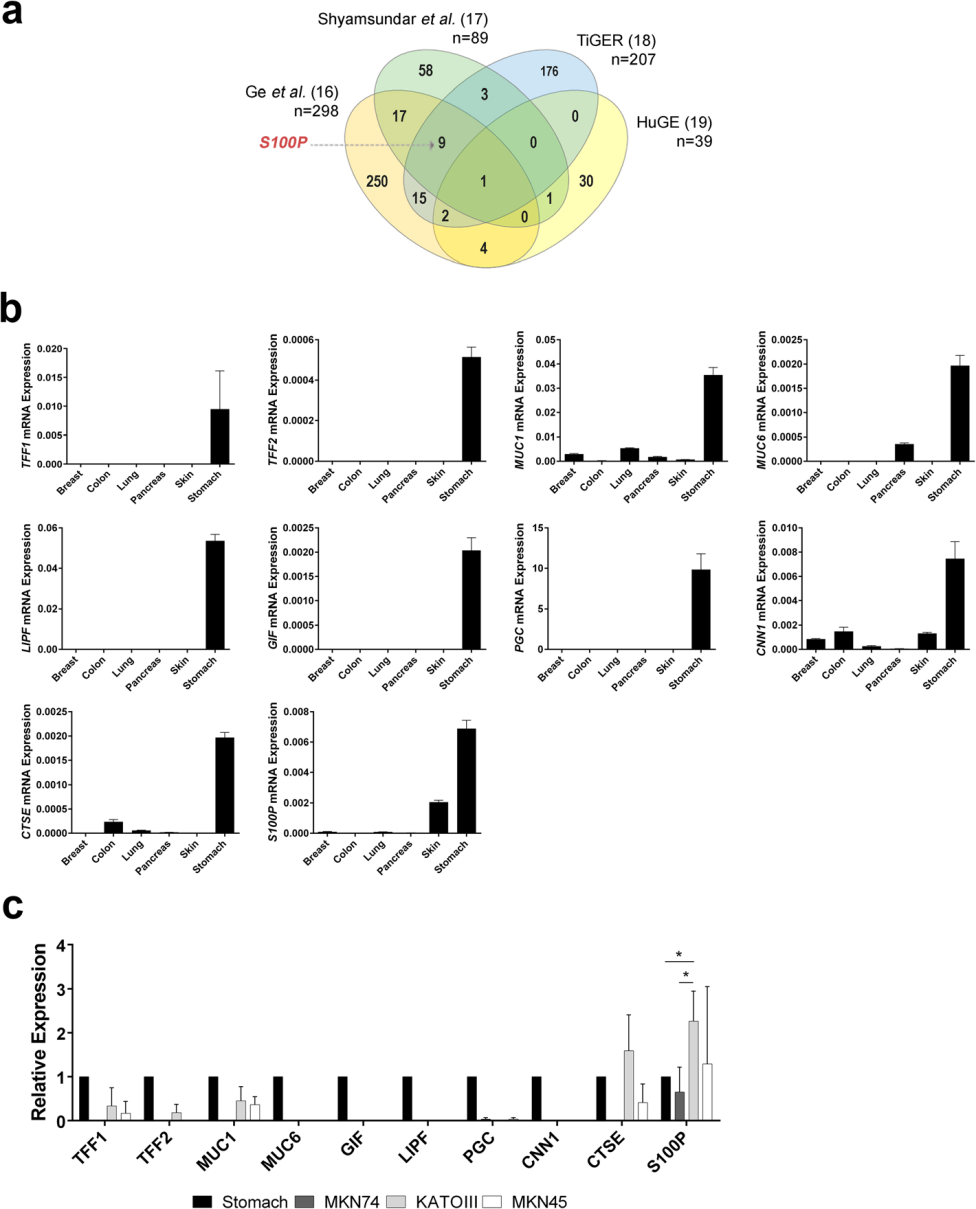
S100P is associated with a gastric-specific signature. **a** Venn Diagram representing the number of genes defined as stomach-specific for each of the 4 datasets used. **b** The expression profile of 10 candidate genes was validated by qRT-PCR in commercial human RNAs representing a pool of at least three different tissue donors. **c** The S100P gene is over-expressed in GC cell lines of the diffuse type with dysfunctional E-cadherin (KATOIII and MKN45). Data represent mean value ±SD of at least three independent experiments normalized to the stomach. Statistical significance was evaluated with the Student’s t-test (*P ≤ 0.05)

Four public datasets were consulted and cross-referenced to define a final list of putative stomach-specific genes: 1) Ge et al dataset consisting of a series of gene expression microarrays performed using 36 human normal tissue types [16]; 2) Shyamsundar et al dataset which defined a list of tissue-specific transcripts [17]; 3) Hsiao et al dataset of a series of gene expression microarrays performed on 59 human samples representing 19 distinct tissue types, stored in the web resource HuGE Index (*http://www.hugeindex.org*) [18]; and 4) the web resource TiGER (tissue-specific gene expression and regulation, which uses several large scale expression datasets allowing the extraction of a tissue-specific gene list [19]). From the Ge et al dataset we selected as stomach-specific genes those whose average expression in stomach was higher than the average expression across all other 35 tissues plus 3 times its standard deviation. This defined a list of 298 genes. From the Shyamsundar et al dataset we extracted the 89 distinct genes defined by the authors as stomach-specific. From the web resource HuGE Index, we selected genes that were detected in stomach and absent in all other tissues analysed, using the query-based search available, which resulted in 39 distinct genes. From the web resource TiGER, we selected the pre-computed list of 207 stomach specific genes. Next, we cross-referenced the four stomach-specific gene lists and defined as the final list the 51 genes detected in 2 or more datasets (Fig. 1a and Additional file 1: Table S1). Using PubMed query-based search and DAVID [20], we determined whether an association of each gene with cell survival, cell apoptosis and GC had already been described (Additional file 1: Table S1).

### Cell culture

Human GC cell lines MKN74, KATOIII, MKN45 and NCI-N87 were obtained from American Type Culture Collection (USA). Cells were routinely cultured at 37 °C in a humidified atmosphere with 5% CO2, in RPMI 1640 (Gibco, Thermo Fisher Scientific, USA) supplemented with 10% foetal bovine serum (Hyclone, Logan, UT) and 1% penicillin–streptomycin (Gibco, Thermo Fisher Scientific, USA).

### Gene silencing by siRNA transfection

All cell lines were seeded in six-well plates for 24 h and transfected using Lipofectamine 2000 (Invitrogen, *Thermo Fisher Scientific, USA*) in serum-free Opti-MEM (Gibco, *Thermo Fisher Scientific, USA*), according to the manufacturer’s recommended procedures. Gene silencing was achieved with ON-TARGET S100P (L-004295-00-0020) and ON-TARGETplus ZEB1 (L-006564-01-0010) from Dharmacon (Lafayette, USA) at a final concentration of 100 nM (optimized to achieve the highest silencing efficiency at the lowest toxicity). A non-targeting siRNA also from Dharmacon (D-001810-01-50; ON-TARGETplus Non-targeting siRNA #1) was used as a negative control. Gene inhibition evaluation and all subsequent assays were performed upon 48 h of cell transfection.

### Gene expression analysis by qRT-PCR

Total RNA from GC cell lines was isolated using the RNA Isolation Kit: RNeasy Mini Kit (Qiagen, Germany). Human total RNA from stomach (HPA540037), pancreas (HPA540023), skin (HPA540031) and breast (HPA540045) was purchased from Agilent Technologies (USA). Human total RNA from colon and lung was obtained from Ambion’s FirstChoice® Human Total RNA Survey Panel (Ambion, Thermo Fisher Scientific, USA). All commercially available human RNAs used represented a pool of at least three tissue donors. Expression levels of CDH1, S100P, TFF1, TFF2, GIF, LIPF, MUC1, MUC6, PGC, CNN1 and CTSE were evaluated by qPCR in cDNA produced with a Superscript III First-Strand Synthesis System (Invitrogen, Thermo Fisher Scientific, USA), according to the manufacturer’s instructions. cDNA used to evaluate expression levels of SNAI1, SNAI2, ZEB1 and ZEB2 was produced using qScript™ cDNA SuperMix, (Quantabio, USA), according to the manufacturer’s instructions. Taqman expression assays for CDH1 (Hs01023894_m1), S100P (Hs00195584_m1), PGC (Hs00160052_m1), MUC1 (Hs00159357_m1), CNN1 (Hs00154543_m1), CTSE (Hs00157213_m1), SNAI1 (Hs00195591_m1), SNAI2 (Hs00161904_m1), ZEB1 (Hs00232783_m1) and ZEB2 (Hs00207691_m1) were purchased from Applied Biosystems. TFF1 (Hs.PT.49a.19108373), TFF2 (Hs.PT.49a.20795479), GIF (Hs.PT.49a.21055412), LIPF (Hs.PT.49a.20206106), and MUC6 (Hs.PT.49a.3931120.g) PrimeTime Mini qPCR Assays were purchased from Integrated DNA Technologies (BVBA). The eukaryotic 18S rRNA (Hs99999901_s1; Applied Biosystems, Thermo Fisher Scientific, USA) was used as an endogenous control gene. Gene expression assays were performed in, at least, three biological replicates using TaqMan® Universal PCR Master Mix, No AmpErase® UNG (Applied Biosystems, Thermo Fisher Scientific, USA) and standard TaqMan thermocycling conditionsin an ABI Prism 7000 Sequence Detection System (Applied Biosystems, Thermo Fisher Scientific, USA). Data were analysed by the comparative 2(−ΔΔCT) method [21].

### Western blotting and antibodies

Protein lysates were prepared from cells in cold Catenin lysis buffer (1% Triton X-100 (Sigma-Aldrich, USA), 1% Nonidet P-40 (Sigma-Aldrich, USA)) in PBS supplemented with a protease inhibitor cocktail (Roche, Switzerland) and a phosphatase inhibitor cocktail (Sigma-Aldrich, USA). Protein concentration was determined using the Bradford assay (BioRad Protein Assay kit, USA) and analysed by Western blotting. Briefly, protein extracts (25 μg/lane) were resolved on 7,5% sodium dodecyl sulphate-polyacrylamide gel electrophoresis (SDS-PAGE) or on 4–20% Mini protean TGX gradient gels (Bio-Rad, USA) under denaturing conditions and transferred to Hybond ECL membranes (Amersham Biosciences, GE Healthcare, UK).

The following primary anti-human antibodies were used: mouse anti-Ecadherin (610,182, BD Biosciences, USA), mouse anti-Ecadherin (clone HECD-1, 13–1700, Thermo Fisher Scientific, USA), goat anti-S100P (AF2957, R&D Systems, USA), rabbit anti-Phospho Akt (Thr308) (2965, Cell Signaling, USA), rabbit anti-Akt (9272, Cell Signaling, USA), rabbit anti-Phopho ERK1/2 (Thr202/Tyr204) (9101, Cell Signaling, USA), anti-ERK1/2 (9102S, Cell Signaling, USA), and anti-TCF8/ZEB1 (D80D3) (3396, Cell Signaling, USA). Mouse anti-α-tubulin (T5168, Sigma-Aldrich, USA) and anti-GAPDH (sc-47,724, Santa Cruz Biotechnology, USA) were used as loading controls.

Anti-rabbit (NA931, GE Healthcare Biosciences, UK), anti-mouse (NA931, GE Healthcare Biosciences, UK) and anti-goat (sc-2020, Santa Cruz Biotechnology, USA) HRP-conjugated secondary antibodies were used, followed by ECL detection (Amersham Biosciences, GE Healthcare, UK). Protein expression differences on immunoblots were quantified using Quantity One 4.6.8 Software (Bio-Rad, USA).

### Flow cytometry analysis

To quantify for cell death, cells were stained with FITC (fluorescein isothiocyanate) Annexin V and PI (FITC Annexin V Apoptosis Detection Kit I; BD Pharmingen, USA) and subjected to flow cytometry analysis, according to the manufacturer’s instructions. Briefly, following 48 h of transfection, cells were detached with Trypsin (Invitrogen, Thermo Fisher Scientific, USA), washed with ice-cold PBS and resuspended in Binding Buffer. Cell suspensions were then incubated with both FITC Annexin V and PI for 20 min in the dark at room temperature. Cells were analyzed in a BD Accuri C6 Flow Cytometer (BD Biosciences, USA). The following controls were used to set up compensation and quadrants: unstained cells; cells stained with FITC Annexin V (no PI); cells stained with PI (no FITC Annexin V). Data was analysed with the BD Accuri C6 Software (Version 1.0.264.21).

### Gastrosphere formation assay

To assess sphere formation, monolayer cells were trypsinized, washed in cold PBS, passed through a 25G needle (3 strokes) and resuspended in serum-free RPMI without phenol red (Gibco, Thermo Fisher Scientific, USA) supplemented with 1% penicillin–streptomycin (Gibco, Thermo Fisher Scientific, USA), 1% N-2 (Gibco, Thermo Fisher Scientific, USA), 2% B-27 (Gibco, Thermo Fisher Scientific, Spain), 10 ng/ml bFGF (Immunotools, Germany) and 20 ng/ml hEGF (Invitrogen, Thermo Fisher Scientific, USA). Cells were plated in 6-well tissue culture plates coated with poly (2-hydroxyethyl methacrylate) (Sigma-Aldrich, USA) at a density of 2000 cells/ml. After 5 days in culture, gastrospheres with a diameter higher than 50 μm were counted using a Leica DMi1 inverted microscope with camera.

### Matrigel invasion assay

Invasion assays were performed in 24-well matrigel invasion chambers (Corning Biocoat Matrigel Invasion Chamber, 8.0 μm PET Membrane, USA) as previously described [22]. Briefly, non-invasive cells were removed with a pre-wet ‘cotton swab’ and invasive cells were fixed in ice-cold methanol for 15 mins followed by mounting on Vectashield with DAPI slides (Vector Laboratories, USA). Invasion was quantified by counting invasive nuclei in a Leica DM2000 microscope.

### Slow aggregation assay

Functionality of cell-cell adhesion complexes was assessed through the slow aggregation assay as previously described [23]. Briefly, cells were trypsinized and then transferred to wells of an agar-coated (0.66% w/v) 96-well plate. Aggregate formation was evaluated at 24 h, 48 h and 92 h using a Leica DMi1 inverted microscope with camera.

### Proximity ligation assay

Cells were cultured on glass coverslips in 6-well plates to at least 80% confluence and fixed in ice-cold methanol for 20 min for both proximity ligation assays (PLA) E-cadherin/b-catenin and E-cadherin/p120. PLA was performed using Duolink Detection kit (Olink Bioscience, Sweden), according to the manufacturer’s instructions for Duolink Blocking solution and Detection protocol. Briefly, slides were blocked, incubated with antibodies directed against E-cadherin cytoplasmic domain (610,182, BD Biosciences or Clone 24E10, #3195, Cell Signaling, USA), b-catenin (C2206, Sigma-Aldrich, USA) and p120 (610,134, BD Biosciences, USA), followed by incubation with the secondary PLA probes (anti-mouse Minus and anti-rabbit Plus) conjugated to unique oligonucleotides. Amplification template oligonucleotides were hybridized to pairs of PLA probe and circularized by ligation. Rolling circle amplification was performed and detection of amplified DNA was possible by addition of complementary oligonucleotides labeled with Cy3 fluorophore. Coverslips were mounted on Vectashield with DAPI (Vector Laboratories, USA). Images were acquired on a Carl Zeiss Apotome Axiovert 200 M Fluorescence Microscope (× 20 and × 40 objectives; Carl Zeiss, Germany) with an Axiocam HRm camera and processed with the Zeiss Axion Vision 4.8 software. Quantification of PLA signals was achieved using BlobFinder V3.2.42.

### Immunofluorescence staining

Cells were cultured to confluent monolayers on glass coverslips and fixed in ice-cold methanol for 20 min, except for S100P stained cells, which were fixed with 4% paraformaldehyde for 20 min. Following a 10 min PBS wash, cells fixed with paraformaldehyde were incubated in NH_4_Cl 50 mM for 10 min, and permeabilized with 0.1% Triton X-100 in PBS for 5 min, at room temperature. Cells were blocked with 3% BSA in PBS and stained with primary antibodies, rabbit anti-S100P (ab133554, Abcam, UK) and mouse anti-E-cadherin (610,182, BD Biosciences, USA), followed by a 1 h incubation in the dark with Alexa 488 or Alexa 594-conjugated secondary IgG (Invitrogen, Thermo Fisher Scientific, USA). Coverslips were mounted with Vectashield with DAPI (Vector Laboratories, USA) and images acquired on a Carl Zeiss Apotome Axiovert 200 M Fluorescence Microscope (× 20 and × 40 objectives; Carl Zeiss, Germany) with an Axiocam HRm camera. Images were processed with the Zeiss Axion Vision 4.8 software.

### Patients

Specimens were collected from all gastric adenocarcinoma patients treated surgically between January 2008 and December 2014 at Centro Hospitalar de São João, Porto, Portugal (*n* = 443), following exclusion of patients with no clinicopathological data available and follow-up losses. Formalin-fixed paraffin-embedded tumour tissue was available from 333 cases, which were included in tissue microarrays (TMAs). All eligible patients provided their written informed consent for use of their tissue. The Ethics Committee of Centro Hospitalar S. João provided ethical approval of the study.

### Immunohistochemistry

S100P and E-cadherin expressions were assessed by imunnohistochemistry in 5 μm sections of FFPE TMAs following standard protocol. Briefly, slides were deparaffinised and hydrated followed by antigen retrieval performed in a HC-Tek Epitope Retrieval Steamer Set for 40 min in 10 mM citrate buffer, pH 6.0. Endogenous peroxidase activity was blocked with 3% hydrogen peroxide for 10 min and primary antibodies (anti-S100P, EPR6143, Abcam, UK and anti-E-Cadherin, Clone 24E10, 3195, Cell Signaling, USA) were incubated overnight. Dako REAL Envision Detection System Peroxidase/DAB+ (DAKO, Denmark) was used for detection and sections were then counterstained with hematoxylin, dehydrated and mounted. An experienced pathologist (FS) performed grading of staining, and cases were dichotomized using a simplified classification scheme: retaining (graded as 3+) versus loss (0 to 2+), or positive (from 1+ to 3+) versus negative (graded as 0).

### Statistical analysis

All experimental assays were carried out with at least three independent biological replicates. Results are expressed as mean ± standard deviation (SD).

Statistical analyses were performed using the unpaired two-tailed t-test in GraphPad Prism (version 6.05).

For TMA statistics, Chi-square or Fisher exact tests were used for comparison of proportions among cases, according to the two biomarkers evaluated. Age factor was evaluated through the Mann-Whitney U test. Patients’ overall and relapse-free survival was calculated using the Kaplan-Meier method and significance of differences between crude survival curves was tested by the log-rank test. All statistical analyses were performed in IBM SPSS Statistics version 24.

## Results

In this study, we aimed to identify gastric specific molecular factors that may influence the expression and function of E-cadherin associated to GC.

### S100P is part of a gastric-specific signature

We first set out to identify the gastric specific signature that may underlie the E-cadherin-associated GC molecular program. A putative stomach-specific gene list was compiled based on data mining analysis of 4 datasets of expression arrays of normal tissues by assessing which genes were present in at least two of those datasets. We obtained 51 common stomach-specific genes, which were then matched to state of the art literature aiming at identifying among them (from normal tissue) those that could be relevant to cell survival/apoptosis and/or GC (Additional file 1: Table S1; Fig. 1a). Amid a list of potential candidates, we selected 10 genes for further analysis based upon their involvement in pathways that could intersect with E-cadherin mediated signaling. We validated the bioinformatic analysis by qRT-PCR for the selected genes and confirmed their expression to be particularly enriched in RNA from stomach tissue, when compared with RNA of other epithelial tissues, namely breast, colon, lung, pancreas and skin (Fig. 1b).

The same set of genes was then evaluated in a panel of GC cell lines. We verified that, in contrast to the other genes, *S100P* was expressed in all GC cell lines tested, namely those displaying either functional (MKN74) or dysfunctional E-cadherin (KATOIII and MKN45) [24]. Further, we observed that cancer cell lines with E-cadherin dysfunction displayed a significant upregulation of S100P expression when compared to those with wild-type E-cadherin and the stomach tissue (Fig. 1c).

### S100P inhibition decreases E-cadherin expression and impairs the assembly of the cadherin-catenin complex

**Fig. 2:**
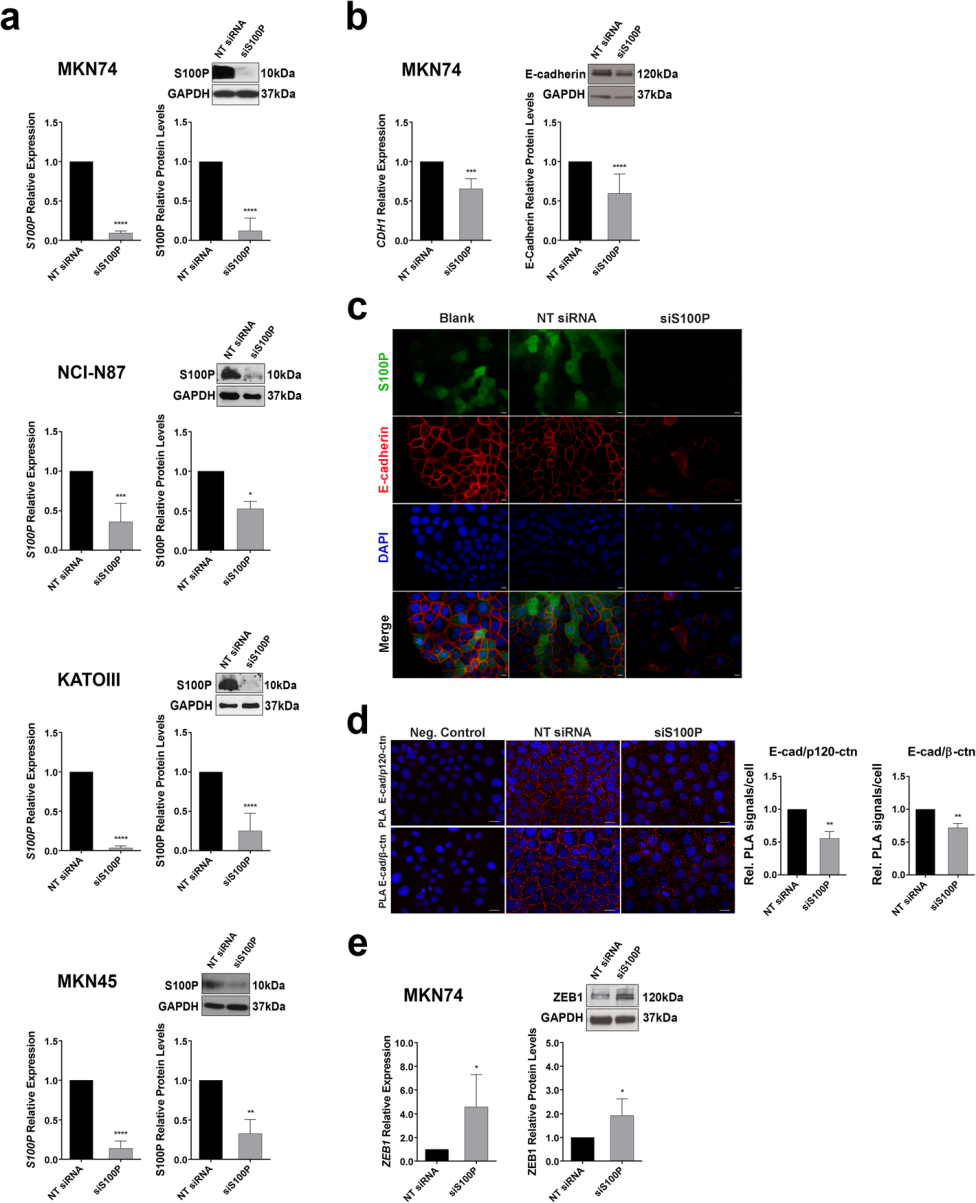
S100P inhibition affects E-cadherin expression via ZEB1 upregulation and hampers its stabilization at the membrane. **a** S100P expression, evaluated by qRT-PCR and Western blot, decreases in MKN74, NCI-N87, KATOIII and MKN45 cells transiently transfected with a siRNA for S100P (siS100P). Transfection with non-targeting siRNA (NT siRNA) was used as control. **b** Transient inhibition of S100P leads to a decrease of E-cadherin expression in MKN74, confirmed by qRT-PCR and Western blot. **c** MKN74 cells transfected with non-targeting siRNA (NT siRNA) or siRNA for S100P (siS100P) were fixed and stained with anti-human S100P antibody (green) and anti-human E-cadherin antibody (red). Nuclei were counterstained with DAPI (blue). Scale bar represents 10 μm. **d** The interaction between E-cadherin and *β*-catenin or *p120*-catenin was analyzed by PLA following S100P silencing by siRNA in MKN74 cells. Red dots indicate PLA signals and nuclei were counterstained with DAPI (blue). Scale bar represents 20 *μ*m. The number of PLA signals per cell was quantified in each condition. **e** ZEB1, a CDH1 transcription repressor, is upregulated at both RNA and protein levels following S100P silencing in MKN74 cells. GAPDH was used as loading control. Data represent relative mean value ±SD and images are illustrative of at least three independent experiments. Statistical significance was evaluated with the Student’s t-test (*P ≤ 0.05; **P ≤ 0.01; ***P ≤ 0.001; ****P ≤ 0.0001)

To evaluate the role of S100P in E-cadherin associated GC, we knocked down its expression by specific small interfering RNA (siRNA) in GC cells expressing either functional (MKN74 and NCI-N87) or dysfunctional E-cadherin (KATOIII and MKN45) (Additional file 2: Table S2 [24, 25];). Upon transfection, silencing of S100P was confirmed by qRT-PCR and Western blot analysis demonstrating that S100P protein expression levels were decreased to near absent levels (Fig. 2a). We first verified that silencing of S100P in wild-type E-cadherin MKN74 cells led to a significant decrease in the expression of E-cadherin, both at the RNA (*p* = 0.0002) and protein (*p* < 0.0001) levels (Fig. 2b). Furthermore, as depicted in Fig. 2c, whereas the control cells display strong membrane staining of E-cadherin, cells depleted of S100P exhibited much lower levels of E-cadherin at the membrane together with a diffuse protein distribution throughout the cytoplasm. Expression levels of E-cadherin upon S100P silencing were also evaluated for the remaining cell lines, demonstrating a tendency of reduced E-cadherin expression in wild-type E-cadherin NCI-N87 (Additional file 3: Figure S1).

Following the evidence that S100P is likely to regulate the levels of E-cadherin, we next validated its impact in the stability of the adhesion complex. The ability to establish a stable cadherin-catenin complex was thus evaluated through PLA. Our data demonstrated that wild-type E-cadherin cells transfected with S100P-specific siRNA exhibited a significant decrease in the interaction between E-cadherin and the adhesion complex members when compared to control cells. Specifically, the amount of interactions, depicted as red blobs, between E-cadherin and *p120-catenin* were reduced from 1.00 to 0.56 in cells depleted of S100P (*p* = 0.0055). Likewise, S100P inhibition led to a reduction from 1.00 to 0.77 in the number of PLA signals resulting from the interplay between E-cadherin and *β-catenin* (*p* = 0.0122), indicating that S100P impacts on E-cadherin membrane stability and disturbs the assembly of the adhesion complex (Fig. 2d).

Together, these results prompted us to investigate the molecular mechanism underlying S100P downregulation of E-cadherin expression, focusing on the expression of E-cadherin transcriptional repressors ZEB1/2, Slug or Snail. S100P inhibition did not affect the expression levels of ZEB2 but led to an increased tendency in the levels of SNAI1 (Snail) and SNAI2 (Slug) mRNA (Additional file 4: Figure S2a). More so, significantly increased levels of both ZEB1 mRNA (1.00 to 4.59, *p* = 0.0405) and protein (1.00 to 1.92, *p* = 0.0368) were observed in MKN74 cells following S100P silencing, revealing that S100P is positively regulating E-cadherin expression through a repression of ZEB1 expression (Fig. 2e).

To further evaluate the role of ZEB1 in S100P-mediated regulation of E-cadherin, we modulated ZEB1 expression levels by siRNA in cells expressing wild-type E-cadherin (MKN74). Our results demonstrate that downregulation of ZEB1 led to an increased tendency in the expression levels of both E-cadherin (1.00 to 1.3) and S100P (1 to 2.28). Remarkably, the effect of ZEB1 downregulation in E-cadherin was abolished upon S100P inhibition, which supports that regulation of E-cadherin expression is S100P dependent (Additional file 4: Figure S2b).

### S100P silencing yields different cellular behaviours depending on the E-cadherin cellular context

**Fig. 3:**
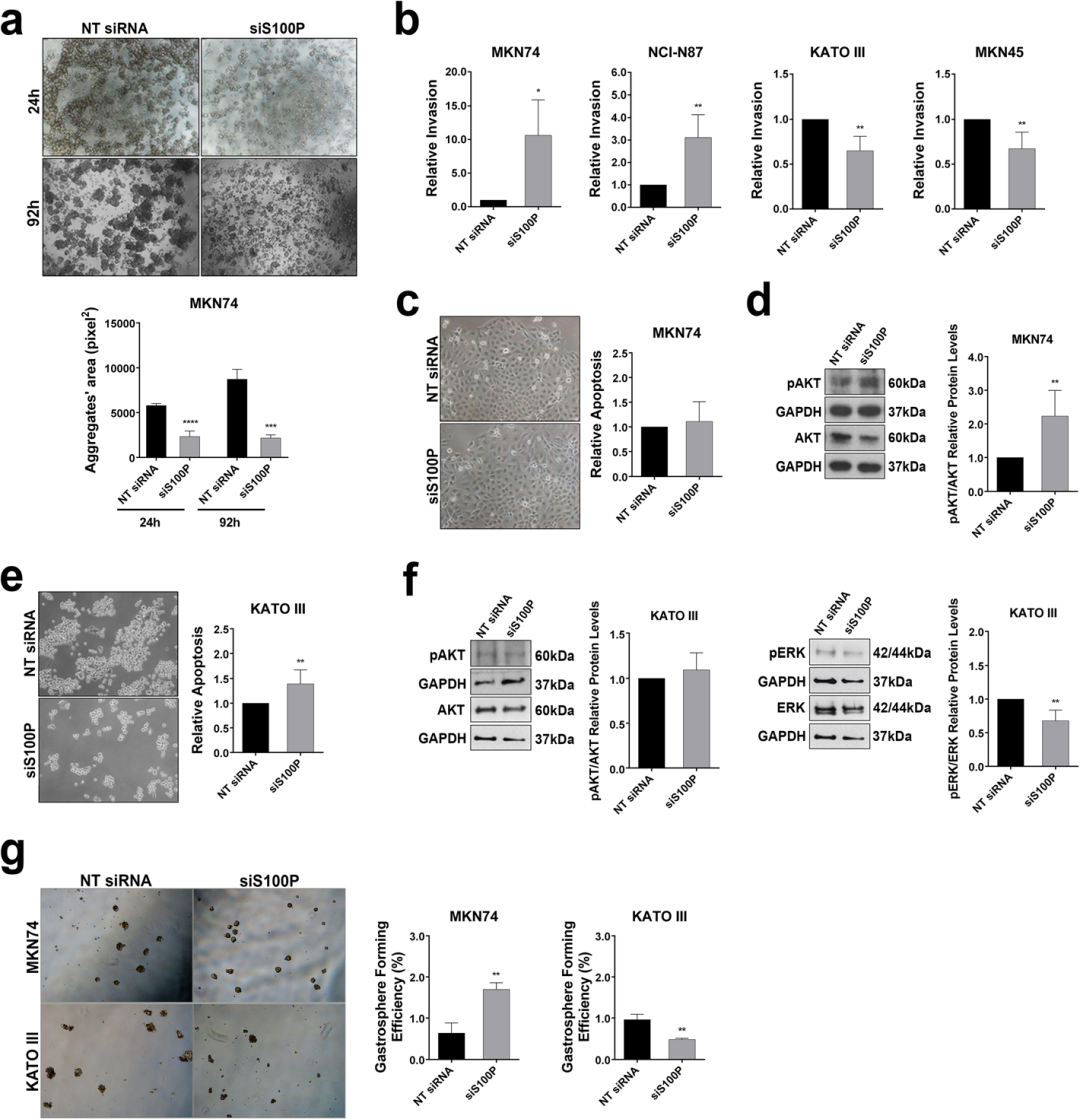
S100P downregulation elicits different cellular behaviours in an E-cadherin dependent manner. **a** Cell-cell adhesion ability, evaluated by the slow aggregation assay, is compromised in MKN74 cells following transfection with siS100P. The graph shows quantification of aggregate area at 24 and 92 h. **b** Matrigel invasion assays were performed for MKN74, NCI-N87, KATOIII and MKN45 cells following transient transfection with siS100P (or with non-targeting siRNA). The graphs depict the relative number of invasive cells ±SD. Upon S100P downregulation, the invasive capacity increases in MKN74 and NCI-N87 cells but decreases in KATOIII and MKN45 cells. Apoptosis was evaluated upon depletion of *S100P* in MKN74 **(c)** and KATOIII **(e)** 48 h post transfection. Briefly, cells were stained with FITC Annexin V and propidium iodide (PI) and relative apoptosis levels were measured by flow cytometry, indicating a significant increase in the levels of apoptotic cells in KATOIII. **d** The levels of phosphorylated AKT were analyzed by Western blot, indicating increased activation in MKN74 cells. **f** Phosphorylated AKT and ERK expressions were evaluated by Western blot in KATOIII cells. Silencing of S100P did not alter AKT expression but led to a decrease in ERK activation. **g** Self-renewal potential, determined by the sphere-formation assay, increases in MKN74 cells and decreases in KATO III upon S100P downregulation. Sphere-forming efficiency is calculated based on the number of spheres divided by the number of cells plated. Data correspond to mean value ±SD and images are representative of at least three independent experiments. Statistical significance was evaluated with the Student’s t-test (*P ≤ 0.05; **P ≤ 0.01; ***P ≤ 0.001; ****P ≤ 0.0001)

Upon establishing the effect of S100P in the regulation of E-cadherin expression, we next investigated the impact of S100P on E-cadherin function. We first assessed cell-cell adhesion using slow aggregation assays. As shown in Fig. 3a, we observed that decreased expression of S100P was sufficient to impair cell-cell adhesion both at 24 h (*p* < 0.0001) and at 92 h (*p* = 0.0006) in an E-cadherin wild-type context (MKN74), indicating that S100P interferes with the adhesive function of E-cadherin. Likewise, S100P interfered with cell-cell adhesion in the NCI-N87cell line, although to a much lower extent. In KATOIII and MKN45 cells (with dysfunctional E-Cadherin), S100P inhibition did not affect aggregate formation given that these cells are unable to mediate cell-cell compaction (Additional file 5: Figure S3).

Since impaired E-cadherin mediated cell adhesion is associated with increased invasive abilities [5, 10], we evaluated the cells invasive behavior, using Matrigel invasion assays, which mimic the basement membrane composition in vitro (Fig. 3b). We verified that S100P silencing led to an increase from 1.00 to 10.00 fold in the number of invasive MKN74 cells expressing wild-type E-cadherin (*p* = 0.0129). The same effect was observed for the NCI-N87 cell line (1.00 to 5.00 fold increase; *p* = 0.0077), which is also an E-cadherin functional model (Fig. 3b). Overall, these results support that S100P regulates E-cadherin expression, and consequently, its function.

Strikingly, in dysfunctional E-cadherin KATOIII cells, S100P inhibition significantly hindered the invasive potential of from (1.00 to 0.65; *p* = 0.0048; Fig. 3b). Similar results were obtained upon S100P silencing in the E-cadherin negative MKN45 cell line (1.00 to 0.75 decrease; *p* = 0.0041; Fig. 3b).

Considering that E-cadherin dysfunction results in increased apoptosis resistance of GC cells [26], we next evaluated cell death (Fig. 3c and e; Additional file 5: Figure S3b). As determined by FITC Annexin V staining, we could observe that, in the E-cadherin proficient MKN74 cell line, S100P inhibition did not affect apoptosis (Fig. 3c). However, analysis of signalling pathways associated to cell survival revealed that the levels of pAKT were significantly increased upon S100P inhibition (*p* = 0.0011; Fig. 3d) in MKN74. Interestingly, in E-cadherin defective KATOIII cells, S100P silencing led to a significant increase in apoptosis (*p* = 0.0105; Fig. 3e). The increased cell death was not accompanied by an alteration in pAKT levels but rather by a significant decrease in the expression of phosphorylated ERK (*p* = 0.0083; Fig. 3f), a MAP kinase known to inhibit apoptosis upon activation [27].

Given the dual effects of S100P in cell survival depending on the E-cadherin context, we next set out to evaluate its effects in sphere formation ability, a surrogate marker of self-renewal and anchorage-independent survival [28]. We verified that, upon inhibition of S100P, wild-type E-cadherin MKN74 cells exhibited a significant increase in the number of formed spheres (3.04 fold, *p* = 0.0032). In contrast, in dysfunctional E-cadherin KATOIII cells, silencing of S100P significantly decreased the gastrosphere forming efficiency (1.00 to 0.516 fold, *p* = 0.0008; Fig. 3g). These results indicate that S100P is crucial for anchorage-independent cell survival in the context of dysfunctional E-cadherin, in a process involving ERK activation.

Thus, our in vitro data provides evidence that, in GC cells expressing E-cadherin, silencing of S100P affects E-cadherin expression and function, induces apoptosis resistance and awards cells with increased invasive abilities. Importantly, in cancer cells with dysfunctional E-cadherin, S100P activates a distinct signaling pathway and its expression worsens the global effects mediated by loss of E-cadherin.

### High S100P expression impacts the prognosis of gastric cancer patients with loss of E-cadherin

**Fig. 4:**
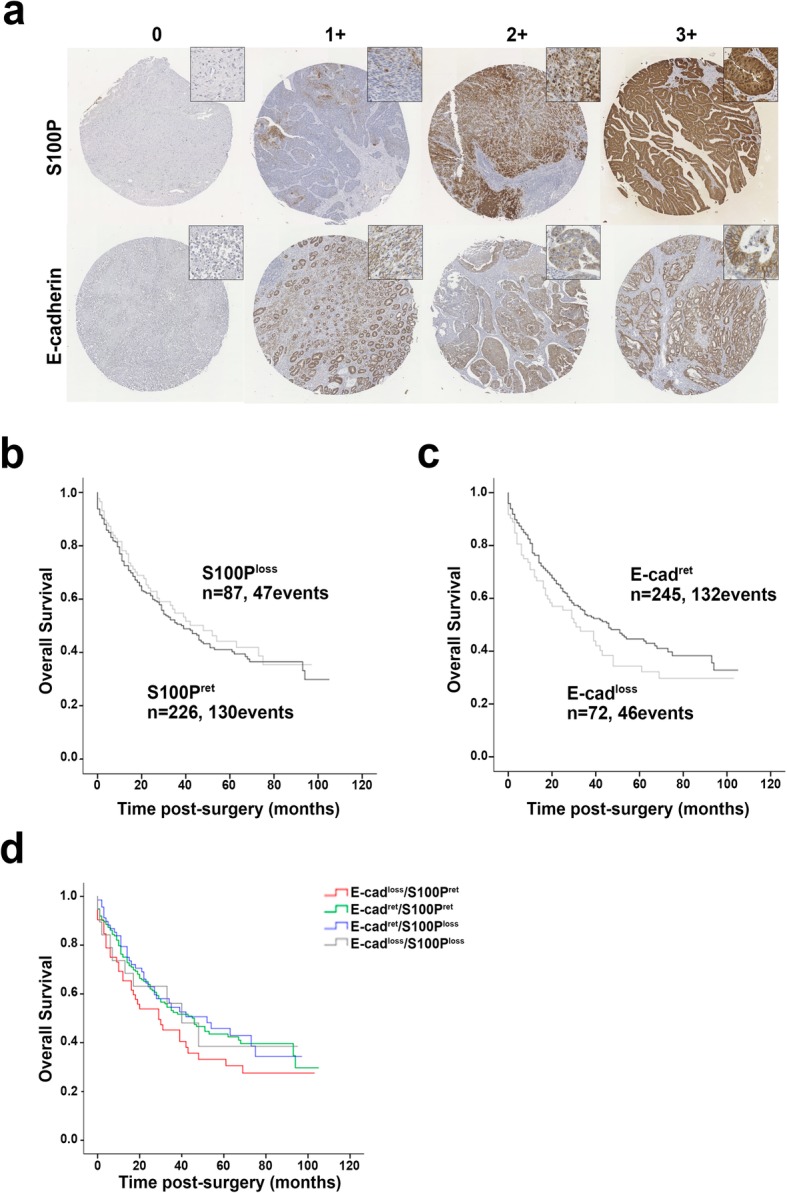
The combination of S100P and E-cadherin molecular profiles defines subgroups with different clinical outcomes. **a** Representative images of S100P and E-cadherin protein expression detected by immunohistochemistry in a gastric carcinoma TMA. Manual annotation of each histological sample was performed by an experienced pathologist and cases were dichotomised using a simplified classification scheme considering retaining (graded as 3+) versus loss of marker from 0 to 2+. **b** Kaplan-Meier curves showing the probability of overall survival for patients with GC according to S100P expression. **c** Kaplan-Meier curves showing the probability of overall survival for patients with GC according to E-cadherin expression, indicating that loss of E-cadherin expression associates with a poorer overall survival. **d** Survival plot depicting the overall survival of patients according to four molecular phenotypes defined by the loss/retention of E-cad and S100P expression. Although not statistically significant, patients presenting the E-cad^loss^/S100P^ret^ molecular phenotype follow a survival curve under that of all other patients

Upon observing distinct functions of S100P in GC models dependent on the E-cadherin functional status, we gathered a single-hospital consecutive GC patient cohort to evaluate the clinical significance of our in vitro results. Immunohistochemical evaluation of both S100P and E-cadherin in a TMA encompassing 333 tumours revealed high expression in 62.7 and 64% of the cases, respectively. Manual annotation of each histological sample was performed by an experienced pathologist and cases were dichotomised using a simplified classification scheme considering retaining (graded as 3+) versus loss for both markers from 0 to 2+ (Fig. 4a). The 5-year overall survival of this cohort was 44% (Additional file 6: Figure S4a). Correlations between S100P and E-cadherin expression, clinicopathological features (Additional file 7: Table S3) and survival analyses demonstrated that S100P expression alone did not predict overall survival (Fig. 4b). On the contrary, loss of E-cadherin expression associated with an overall poorer survival, with a median survival of 16 months for patients with loss of E-cadherin expression versus 43 months for patients with tumours retaining E-cadherin expression (*p* = 0.058; Fig. 4c). However, when we evaluated patients outcomes according to the molecular phenotypes defined by the expression of both proteins, the presence of S100P impacted negatively on the outcome of patients with loss of E-cadherin. Indeed, following a strong tendency towards statistical significance, patients harbouring tumours with the E-cad^loss^/S100P^ret^ molecular phenotype exhibited the worst survival of the cohort (*p* = 0.055; Fig. 4d). Specifically, patients with loss of E-cadherin and retaining S100P expression had a median overall survival of 29 months whereas that of patients bearing tumours with loss of both proteins (E-cad^loss^/S100P^loss^) increased to 40 months.

Given our in vitro results concerning the increased cell’s invasive capabilities upon S100P inhibition in the context of wild-type E-cadherin, we evaluated the effect of S100P^+^ (graded as 3+, 2+, 1+) versus S100P^−^ (graded as 0) in relapse-free survival among the better prognostic group of patients with E-cad^+^ tumours. Supporting the relevance of our data, patients displaying the E-cad^+^/S100P^−^ molecular phenotype had a lower disease-free survival rate than E-cad^+^/S100P^+^ patients (59% versus 65% of patients, respectively), although these results did not reach statistical significance (Additional file 6: Figure S4b).

Overall, our results demonstrate that S100P modulation has different functional and clinical consequences depending on the E-cadherin functional context. Further, in patients bearing E-cadherin negative tumours, S100P expression aggravates patients outcomes, namely their overall survival, indicating that our candidate molecule would be a good therapeutic target for these patients.

## Discussion

This study identifies S100P as a novel molecular determinant of E-cadherin function in GC providing critical information for the management of patients harbouring E-cadherin associated tumours. Although E-cadherin is well recognised as a broad-acting tumour suppressor in hereditary and sporadic gastric carcinomas, we are still far from understanding the molecular events underlying the onset of cadherin-dependent cancer in the gastric epithelium. Thus, the identification of gastric-specific E-cadherin interactors would shed light on the complexity of mechanisms regulating E-cadherin and in the onset of GC. By integrating multiple expression datasets of normal tissues, we could define a list of stomach-specific candidate genes that we postulated could be involved in the aetiology of E-cadherin associated GC. The small calcium-binding protein S100P appeared as a key candidate upon expression analyses on tissues from different epithelial origins. In fact, S100P was specifically expressed in the normal gastric epithelium but not in breast or colon. Moreover, we observed distinct expression levels in GC cell lines displaying either functional or dysfunctional E-cadherin, with the latter displaying higher levels of S100P, supporting an association between S100P and E-cadherin function.

In the majority of cancers, S100P has been evaluated as a potential biomarker for detection of disease and a potential target for therapeutic intervention given its absence in the respective healthy tissues [12, 13]. Functional implications of S100P in the carcinogenic process include effects on cell survival, chemoresistance, invasion and migration [12]. Reports in breast, colon, lung and pancreatic cancers award S100P a key role in cancer initiation predictive of metastatic progression and poor prognosis [12, 29–32]. The fact that S100P is expressed in the adult normal stomach tissue adds complexity to its involvement in gastric tumorigenesis [15]. Although a few reports demonstrate that S100P overexpression may play an oncogenic role in GC, namely by promoting survival and increasing drug resistance of tumour cells [33, 34], conflicting results have also been reported highlighting a putative role of S100P in contributing to sensitization of GC cells to oxaliplatin [35].

This study uncovers dual roles of S100P in the gastric context, which are dependent on the E-cadherin status. In the setting of wild-type E-cadherin, we report a novel mechanism wherein S100P regulates E-cadherin expression contributing to its stabilization at the membrane. Downregulation of S100P in GC cell lines expressing functional E-cadherin affected E-cadherin expression, disturbing the assembly of the cadherin-catenin complex. Further functional studies revealed that knockdown of S100P in wild-type E-cadherin GC cells impaired cell-cell adhesion and promoted both cell’s invasive capacity and sphere formation ability, indicating that S100P interferes with the adhesive and tumour suppressive functions of E-cadherin.

In order to explore the mechanism through which S100P was affecting E-cadherin expression, we evaluated the involvement of relevant transcriptional repressors of the Snail/Slug superfamily. Amongst these, ZEB-1 is one of the transcription factors known to downregulate E-cadherin expression. Binding of ZEB1 to the E-box domain and subsequent downregulation of E-cadherin during epithelial to mesenchymal transition has been thoroughly associated with invasion and metastasis in several cancers [36]. In this study, we observed that S100P inhibition was accompanied by an upregulation of ZEB1 expression that correlated with E-cadherin downregulation and dysfunction. By modulating ZEB1 expression levels, we provided further evidence that E-cadherin and S100P are involved in a common regulatory pathway.

Another key finding of this study was that S100P contributes to an oncogenic molecular program in the stomach by promoting survival of E-cadherin negative GC cells. S100P inhibition promoted cell death and significantly suppressed cells sphere formation ability, awarding a role of S100P in anchorage-independent cell survival of GC cells devoid of E-cadherin. Furthermore, we observed that S100P potentiates the invasive capacity of cells associated to loss of E-cadherin corroborating its oncogenic nature in GC. Our findings are consistent with those of Zhang and colleagues who reported that S100P knockdown promoted cell apoptosis and inhibited colony formation-ability of GC cells [34]. Even though the authors did not address the relationship between S100P function and the E-cadherin status, the gastric cell models used therein are also negative for E-cadherin expression [37], corroborating our results.

In an attempt to dissect the molecular mechanisms underlying S100P activity and function in GC, we assessed the role of S100P in activating signalling pathways previously reported to impact in the development and progression of multiple cancers [15]. Among the signalling pathways where S100P participates, ERK1/2, NF-kB and PI3K/AKT are those most reported [38]. Herein, we demonstrated that the pro-survival effect of S100P on GC cells expressing dysfunctional E-cadherin is associated to ERK activation, which was decreased upon S100P inhibition alongside with increased cell death. In accordance, previous reports have demonstrated that addition of exogenous S100P increased cell survival with simultaneous ERK activation [38]. Interestingly, in the E-cadherin proficient cell line, S100P inhibition did not impact on cell survival and, in fact, we observed a significant increase in AKT activation, a recognized critical regulator of cell survival, which is compatible with a possible compensatory mechanism [39, 40].

Clinical validation of our observations in a TMA of a GC patient cohort revealed that combining S100P and E-cadherin expressions allows the stratification of patients into subgroups with different clinical outcomes. Our results indicate that loss of E-cadherin expression alone correlates with an overall poorer survival corroborating previous reports regarding E-cadherin prognostic value in GC [41]. In contrast, when we evaluated the S100P molecular profile with clinicopathological parameters, we could not award a prognostic value to S100P expression on its own, contradicting a few studies that claim its association with shorter overall survival, GC stage and chemoresistance [33, 34]. However, there are conflicting reports on the prognostic value of S100P in GC [42, 43]. This inconsistency may result from studies that address only the clinical value of S100P without considering other molecular markers, namely the E-cadherin functional status such as we performed in our analysis. Moreover, differences between cancer models may also arise as S100P can act extracellular and/or intracellularly, crosstalking with a variety of signaling pathways, which can be differently activated depending on the tumour origin [38, 44]. Thus, it would be extremely valuable to determine the E-cadherin status of patients in cohorts from previous studies as well as in different cancer models. In our work, we identify distinct effects of S100P on the outcome of patients when we combined S100P molecular profiles to those of E-cadherin. In the subset of tumours expressing E-cadherin, patients with loss of S100P displayed poorer disease-free survival when compared to those expressing S100P. Despite that our results did not reach statistical significance due to the low number of patients exhibiting the E-cad^+^/S100P^−^ molecular phenotype in this cohort, it is very interesting to verify that this data fits our in vitro observations.

Strikingly, within the group of patients bearing E-cadherin negative tumours, those expressing S100P had the worst prognosis of the cohort, substantiating our cellular studies and supporting its oncogenic role as previously suggested for a number of cancers, including GC [12].

Certainly, our observations require validation in larger cohorts and it would also be very relevant to evaluate patient treatment responses in the different molecular profile groups defined by the expression of both S100P and E-cadherin.

## Conclusions

We have identified a novel molecular determinant of E-cadherin function in GC. We demonstrated that S100P inhibition yields distinct cellular effects depending on the E-cadherin functional context. Despite that S100P expression is not an independent prognostic factor, we could identify different prognosis subgroups defined by the combined expression analysis of S100P and E-cadherin. Ultimately, we propose that S100P targeting therapies may benefit the subgroup of patients bearing E-cadherin negative tumours.

## Supplementary information

**Additional file 1: Table S1.** List of stomach-specific genes analysed and their association with cell survival/apoptosis and GC.

**Additional file 2: Table S2.** E-cadherin status and properties of cell lines.

**Additional file 3: Figure S1.** GC cells expressing either functional (NCI-N87) or dysfunctional E-cadherin (KATOIII and MKN45) were transfected with non-targeting sirNA (NT siRNA) or siRNA for S100P (siS100P). E-cadherin expression levels were confirmed by qRT-PCR and Western blot. Data represent mean value ±SD of at least three independent experiments normalized to the control. Statistical significance was evaluated with the Student’s t-test.

**Additional file 4: Figure S2.** a. E-cadherin repressors SNAIl and SNAI2 are upregulated upon SlOOP inhibition. Expression was evaluated by qRT-PCR and 18s was used as loading control. b. Transient inhibition of ZEB1 leads to increased expression levels of both CDH1 and S100R GC cells expressing functional E-cadherin (MKN74) were transfected with non-targeting siRNA (NT siRNA), siRNA for ZEB1 (siZEB1) alone and in combination with siRNA for S100P (siSlOOP/siZEB1). mRNA expression of CDH1, SlOOP and ZEB1 was evaluated by qRT-PCR. Data represent mean value ±SD of at least three independent experiments normalized to the control. Statistical significance was evaluated with the Student’s t-test (*P≤0.05; **P≤001; ***P≤0 001).

**Additional file 5: Figure S3.** a. Cell-cell adhesion ability, evaluated by the slow aggregation assay reveals smaller aggregates in NCI-N87 cells following transfection with siSlOOP. In contrast, cell-cell adhesion ability was neither affected in KATOIII nor in MKN45 cells upon silencing of SlOOP. The images shown are representative of three independent experiments at two time points (24h, 48h or 92h). b. Apoptosis was evaluated upon depletion of SlOOP in NCI-N87 and MKN45 cells 48h post transfection. Briefly, cells were stained with FITC Annexin V and propidium iodide (P1) and relative apoptosis levels were measured by flow cytometry. Statistical significance was evaluated with the Student’s t-test.

**Additional file 6: Figure S4.** a. Kaplan-Meier curve illustrating the probability of overall survival for patients within the study cohort (*n*=333). b. Kaplan-Meier curves showing the probability of relapse-free survival for GC patients according to the molecular phenotypes E-cad/S1OOP and E-cad/S100P. Among the better prognostic group of patients with E-cad tumours, loss of SlOOP defines a worse prognosis subgroup.

**Additional file 7: Table S3.** Summary of GC clinicopathological parameters according to the expression of S100P and E-Cadherin.

## Data Availability

The datasets supporting the conclusions of this article are available at: Ge et al dataset: https://www.ebi.ac.uk/arrayexpress/experiments/E-GEOD-2361/ Shyamsundar et al. dataset: https://www.ebi.ac.uk/arrayexpress/experiments/E-GEOD-2194/?query=+Shyamsundar Hsiao et al dataset: *http://www.hugeindex.org* TIGER web resource: http://bioinfo.wilmer.jhu.edu/tiger/

## Abbreviations

DAPI: 4′,6-diamidino-2-phenylindole
E-cad: E-cadherin
FITC: Fluorescein isothiocyanate
GC: Gastric cancer
PLA: *Proximity ligation assay*
SD: Standard deviation
siRNA: Small interfering RNA
TMA: Tissue microarray
