# Supplementary material for: S100P is a molecular determinant of E-cadherin function in gastric cancer
Source: Cell Commun Signal. 2019 Nov 25;17:155. doi: 10.1186/s12964-019-0465-9 (PMC6878717; doi:10.1186/s12964-019-0465-9)
Supplement: Supplementary file 1 — Additional file 1: Table S1. List of stomach-specific genes analysed and their association with cell survival/apoptosis and GC. [file 12964_2019_465_MOESM1_ESM.docx]

**Table S1.** List of stomach-specific genes analysed and their association with cell survival/apoptosis and gastric cancer.

| **Gene Name** | **Datasets** | | | | **#Datasets** | **Association with** | |
| --- | --- | --- | --- | --- | --- | --- | --- |
|  | Ge *et al.* (16) | Shyamsundar *et al.* (17) | TiGER web resource (18) | HuGE web resource (19) |  | Cell survival/apoptosis | Gastric Cancer |
| *ADAM28* | 1 | 0 | 1 | 0 | 2 | yes | not found |
| *AGR2* | 1 | 1 | 1 | 0 | 3 | yes | yes |
| *AKR1B10* | 1 | 1 | 0 | 0 | 2 | yes | yes |
| *ANKRD49* | 1 | 0 | 1 | 0 | 2 | yes | yes |
| *ANXA10* | 1 | 1 | 1 | 0 | 3 | yes | yes |
| *ARL14* | 1 | 0 | 1 | 0 | 2 | not found | not found |
| *ATP4A* | 1 | 0 | 0 | 1 | 2 | yes | yes |
| *ATP4B* | 1 | 0 | 0 | 1 | 2 | not found | yes |
| *CA2* | 1 | 1 | 0 | 0 | 2 | yes | yes |
| *CA9* | 1 | 1 | 0 | 0 | 2 | not found | yes |
| *CAPN9* | 1 | 1 | 0 | 0 | 2 | not found | yes |
| *CASP10* | 1 | 0 | 1 | 0 | 2 | yes | yes |
| *CCKBR* | 1 | 0 | 0 | 1 | 2 | not found | yes |
| *CDC42EP1* | 1 | 1 | 1 | 0 | 3 | not found | not found |
| *CLDN18* | 1 | 1 | 1 | 0 | 3 | not found | yes |
| *CNN1* | 0 | 1 | 1 | 0 | 2 | yes | not found |
| *CSF2* | 1 | 1 | 0 | 0 | 2 | yes | not found |
| *CTSE* | 1 | 1 | 1 | 1 | 4 | yes | yes |
| *DGKD* | 1 | 1 | 0 | 0 | 2 | not found | not found |
| *EPS8L3* | 0 | 1 | 1 | 0 | 2 | not found | not found |
| *ERN2* | 1 | 0 | 1 | 0 | 2 | not found | not found |
| *FER1L4* | 1 | 1 | 0 | 0 | 2 | not found | yes |
| *FUT1* | 1 | 1 | 0 | 0 | 2 | not found | yes |
| *GALNT3* | 1 | 0 | 1 | 0 | 2 | not found | not found |
| *GATA6* | 1 | 0 | 1 | 0 | 2 | yes | yes |
| *GCNT1* | 1 | 0 | 1 | 0 | 2 | not found | not found |
| *GIF* | 1 | 0 | 1 | 0 | 2 | not found | yes |
| *GKN1* | 1 | 0 | 1 | 0 | 2 | yes | yes |
| *GPX2* | 1 | 1 | 0 | 0 | 2 | yes | yes |
| *IGFBP2* | 1 | 1 | 0 | 0 | 2 | yes | yes |
| *KCNQ1* | 1 | 0 | 0 | 1 | 2 | not found | yes |
| *KLK11* | 1 | 1 | 0 | 0 | 2 | not found | yes |
| *LIPF* | 1 | 0 | 1 | 0 | 2 | not found | yes |
| *LSR* | 1 | 0 | 1 | 0 | 2 | not found | yes |
| *MAFK* | 1 | 0 | 1 | 0 | 2 | not found | not found |
| *MST1R* | 1 | 1 | 0 | 0 | 2 | not found | yes |
| *MUC1* | 1 | 1 | 1 | 0 | 3 | yes | yes |
| *MUC5AC* | 1 | 1 | 1 | 0 | 3 | not found | yes |
| *MUC6* | 1 | 0 | 1 | 1 | 3 | not found | yes |
| *NQO1* | 1 | 1 | 0 | 0 | 2 | yes | yes |
| *PGC* | 1 | 1 | 0 | 0 | 2 | yes | yes |
| *PIK3C2G* | 1 | 1 | 0 | 0 | 2 | not found | not found |
| *PSCA* | 1 | 1 | 0 | 0 | 2 | not found | yes |
| *RECQL5* | 1 | 1 | 0 | 0 | 2 | yes | not found |
| *S100P* | 1 | 1 | 1 | 0 | 3 | yes | yes |
| *SLC4A2* | 1 | 1 | 0 | 0 | 2 | not found | yes |
| *SST* | 0 | 1 | 0 | 1 | 2 | yes | yes |
| *SULT1C1* | 0 | 1 | 1 | 0 | 2 | not found | not found |
| *TFF1* | 1 | 1 | 1 | 0 | 3 | yes | yes |
| *TFF2* | 1 | 0 | 1 | 1 | 3 | yes | yes |
| *TSPAN8* | 1 | 0 | 1 | 0 | 2 | yes | yes |
