# Supplementary material for: S100P is a molecular determinant of E-cadherin function in gastric cancer
Source: Cell Commun Signal. 2019 Nov 25;17:155. doi: 10.1186/s12964-019-0465-9 (PMC6878717; doi:10.1186/s12964-019-0465-9)
Supplement: Supplementary file 2 — Additional file 2: Table S2. E-cadherin status and properties of cell lines. [file 12964_2019_465_MOESM2_ESM.docx]

**Table S2.** E-cadherin status and properties of cell lines.

| **Cell Line** | **Cell Bank** | **E-cadherin Mutation Status** | **mRNA Transcripts** | **Protein** | **Localization** |
| --- | --- | --- | --- | --- | --- |
| **MKN74** | JCRB0255 MKN74 | Wild type | Full-length | Present | Membrane |
| **NCI-N87** | ATCC^®^ CRL-5822^™^ | Wild type | Full-length | Present | Membrane |
| **KATO III** | ATCC^®^ HTB-103^™^ | G to A base substitution of the last 3´nucleotide of exon 7 | 4 alternative | Present | Cytoplasm |
| **MKN45** | JCRB0254 MKN45 | 18-b.p. deletion in exon 6 –intron 6 boundary | 12-bp in-frame deletion | Present | Cytoplasm |
