# Supplementary figures and images for: S100P is a molecular determinant of E-cadherin function in gastric cancer
Source: Cell Commun Signal. 2019 Nov 25;17:155. doi: 10.1186/s12964-019-0465-9 (PMC6878717; doi:10.1186/s12964-019-0465-9)

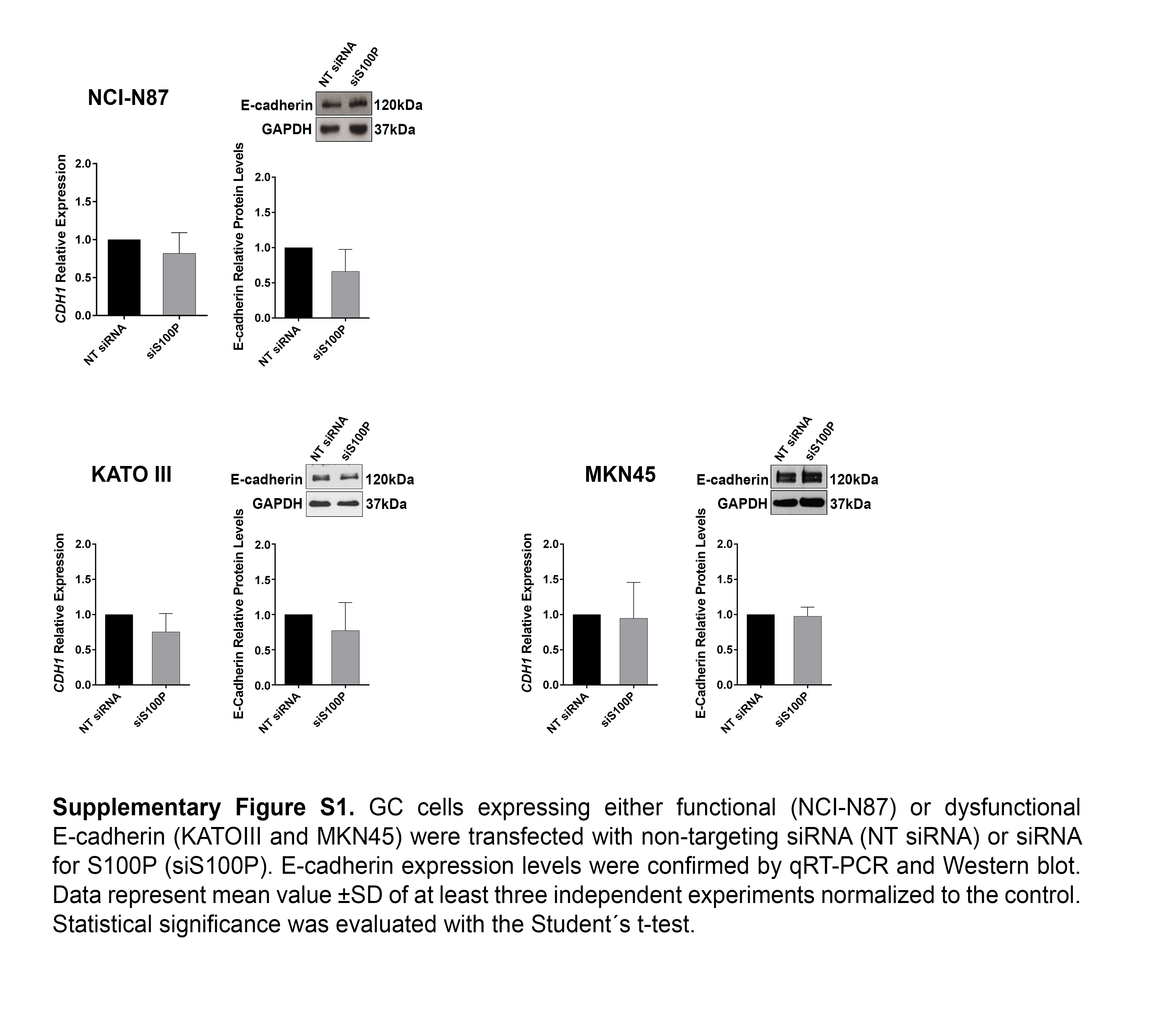

Supplement: Supplementary file 3 — Additional file 3: Figure S1. GC cells expressing either functional (NCI-N87) or dysfunctional E-cadherin (KATOIII and MKN45) were transfected with non-targeting sirNA (NT siRNA) or siRNA for S100P (siS100P). E-cadherin expression levels were confirmed by qRT-PCR and Western blot. Data represent mean value ±SD of at least three independent experiments normalized to the control. Statistical significance was evaluated with the Student’s t-test. [file 12964_2019_465_MOESM3_ESM.tif]

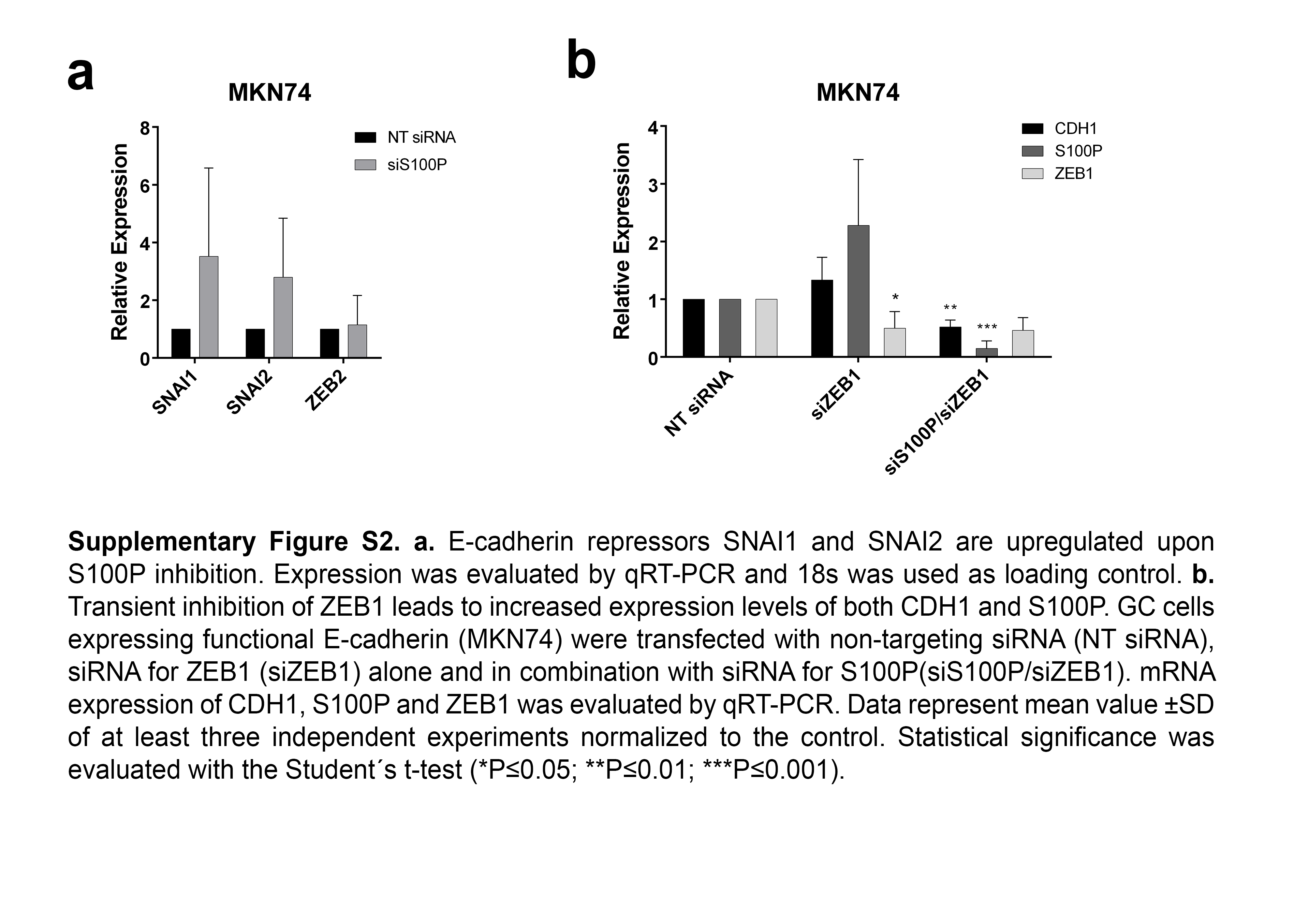

Supplement: Supplementary file 4 — Additional file 4: Figure S2. a. E-cadherin repressors SNAIl and SNAI2 are upregulated upon SlOOP inhibition. Expression was evaluated by qRT-PCR and 18s was used as loading control. b. Transient inhibition of ZEB1 leads to increased expression levels of both CDH1 and S100R GC cells expressing functional E-cadherin (MKN74) were transfected with non-targeting siRNA (NT siRNA), siRNA for ZEB1 (siZEB1) alone and in combination with siRNA for S100P (siSlOOP/siZEB1). mRNA expression of CDH1, SlOOP and ZEB1 was evaluated by qRT-PCR. Data represent mean value ±SD of at least three independent experiments normalized to the control. Statistical significance was evaluated with the Student’s t-test (*P≤0.05; **P≤001; ***P≤0 001). [file 12964_2019_465_MOESM4_ESM.tif]

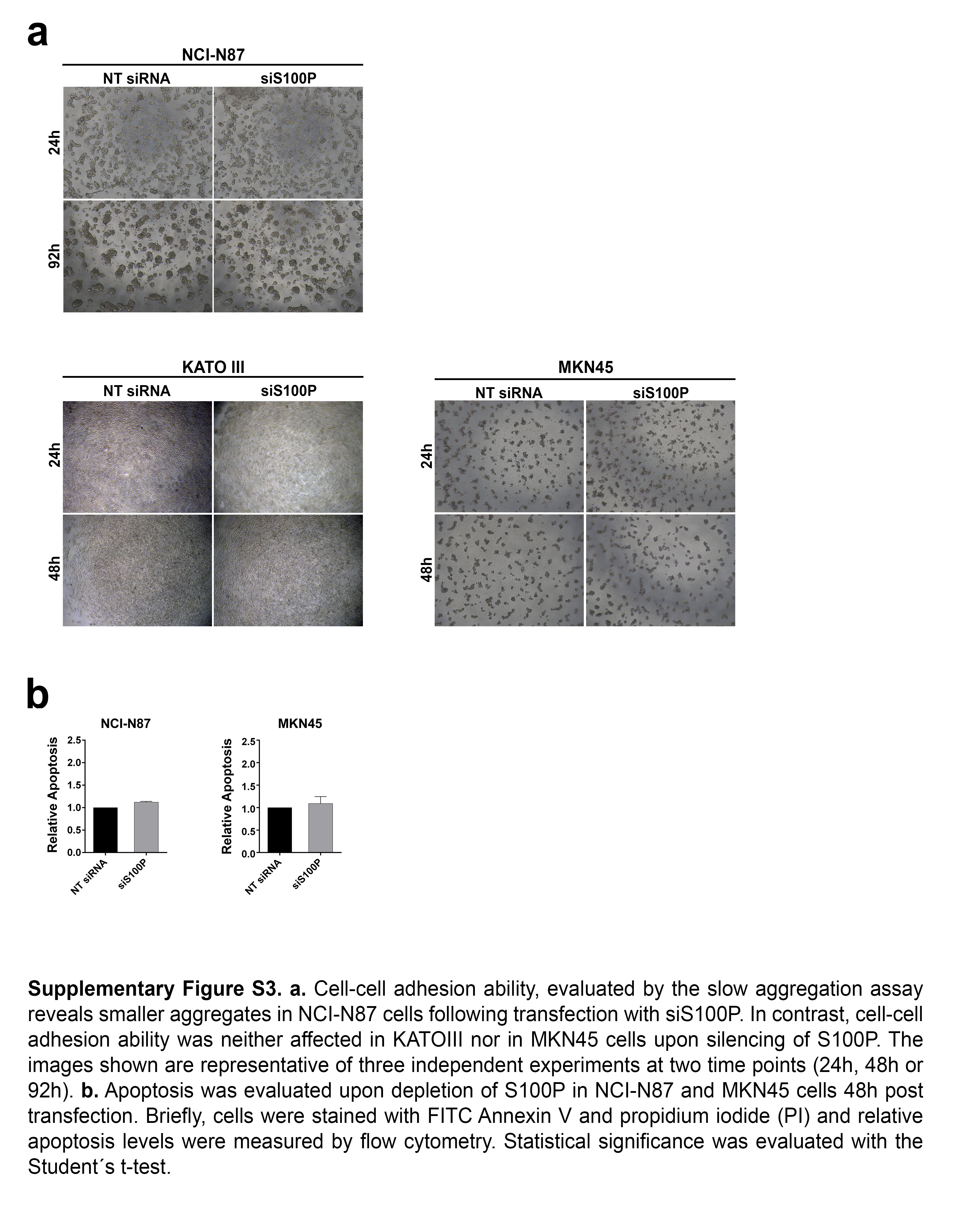

Supplement: Supplementary file 5 — Additional file 5: Figure S3. a. Cell-cell adhesion ability, evaluated by the slow aggregation assay reveals smaller aggregates in NCI-N87 cells following transfection with siSlOOP. In contrast, cell-cell adhesion ability was neither affected in KATOIII nor in MKN45 cells upon silencing of SlOOP. The images shown are representative of three independent experiments at two time points (24h, 48h or 92h). b. Apoptosis was evaluated upon depletion of SlOOP in NCI-N87 and MKN45 cells 48h post transfection. Briefly, cells were stained with FITC Annexin V and propidium iodide (P1) and relative apoptosis levels were measured by flow cytometry. Statistical significance was evaluated with the Student’s t-test. [file 12964_2019_465_MOESM5_ESM.tif]

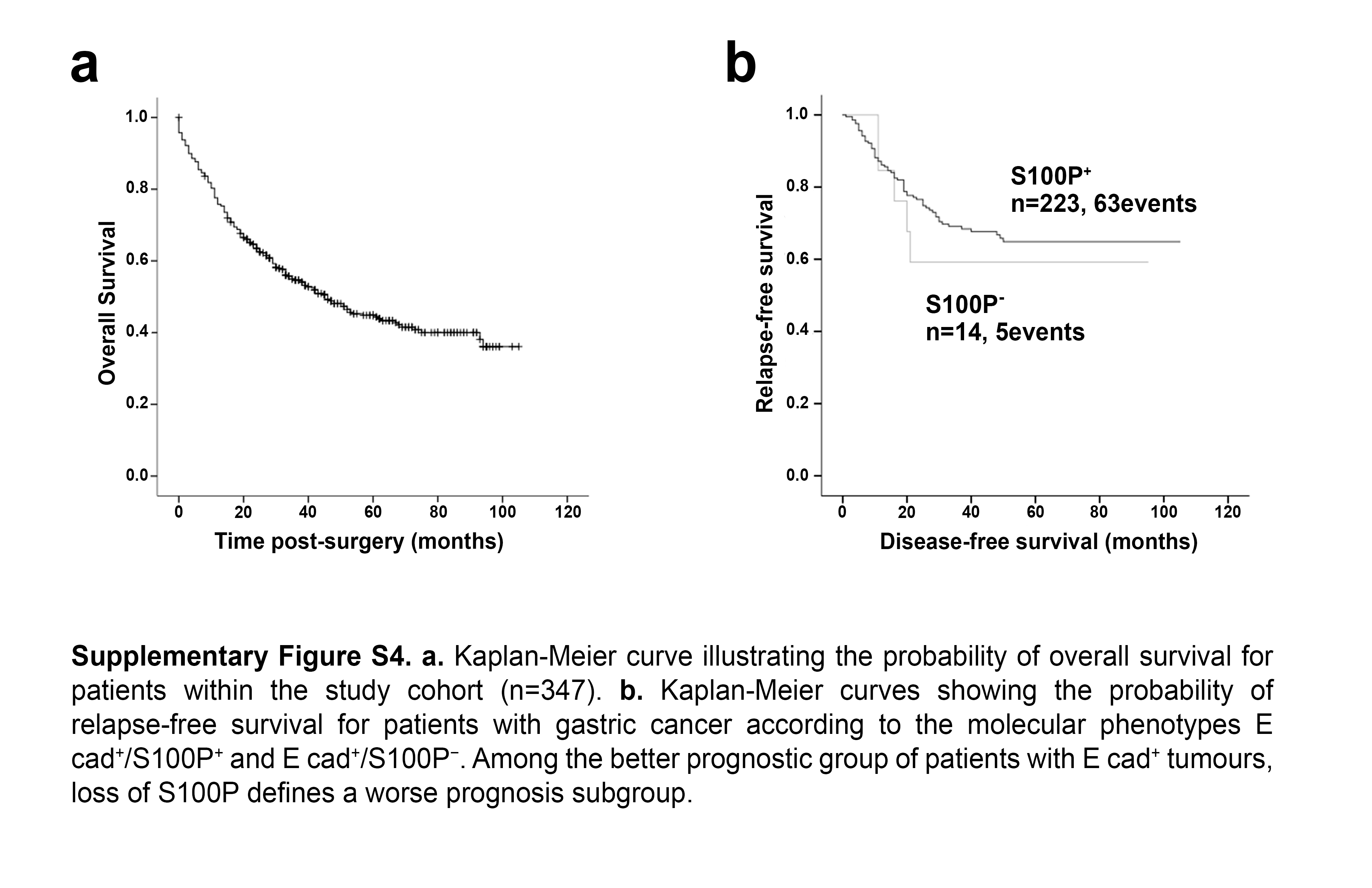

Supplement: Supplementary file 6 — Additional file 6: Figure S4. a. Kaplan-Meier curve illustrating the probability of overall survival for patients within the study cohort (n=333). b. Kaplan-Meier curves showing the probability of relapse-free survival for GC patients according to the molecular phenotypes E-cad/S1OOP and E-cad/S100P. Among the better prognostic group of patients with E-cad tumours, loss of SlOOP defines a worse prognosis subgroup. [file 12964_2019_465_MOESM6_ESM.tif]
