# Supplementary material for: S100P is a molecular determinant of E-cadherin function in gastric cancer
Source: Cell Commun Signal. 2019 Nov 25;17:155. doi: 10.1186/s12964-019-0465-9 (PMC6878717; doi:10.1186/s12964-019-0465-9)
Supplement: Supplementary file 7 — Additional file 7: Table S3. Summary of GC clinicopathological parameters according to the expression of S100P and E-Cadherin. [file 12964_2019_465_MOESM7_ESM.docx]

**Table S3.** Summary of the clinicopathological parameters of gastric cancer according to the expression of S100P and E-Cadherin.

|  | **S100P** | | | | |  | **E-Cadherin** | | | | |
| --- | --- | --- | --- | --- | --- | --- | --- | --- | --- | --- | --- |
|  | ***loss*** | | ***retained*** | |  |  | ***loss*** | | ***retained*** | |  |
|  | **N** | % | **N** | % | *p* |  | **N** | % | **N** | % | *p* |
| Patients* | **87** | 26,9 | **231** | 71,5 |  |  | **72** | 22,3 | **250** | 77,7 |  |
| Age, years | | | | | | | | | | | |
| Median (range) | 68 (32-95) | | 70 (32-87) | | 0,60 |  | 66 (32-95) | | 70,5 (32-92) | | 0,004 |
| Sex | | | | | | | | | | | |
| Male | **60** | 69.0 | **121** | 52,4 | 0,005 |  | **37** | 51,4 | **146** | 58,4 | 0,17 |
| Female | **27** | 31,0 | **110** | 47,6 |  |  | **35** | 48,6 | **104** | 41,6 |  |
| *WHO classification* | | | | | | | | | | | |
| Papillary | **1** | 1,1 | **1** | 0,4 | 0,065 |  | **0** | 0,0 | **2** | 0,8 | <0,001 |
| Tubular | **46** | 52,9 | **86** | 37,6 |  |  | **11** | 15,3 | **124** | 50 |  |
| Mucinous | **1** | 1,1 | **6** | 2,6 |  |  | **0** | 0,0 | **6** | 2,4 |  |
| Poorly cohesive | **10** | 11,5 | **25** | 10,9 |  |  | **21** | 29,2 | **14** | 5,6 |  |
| Other variants | **29** | 33,3 | **111** | 48,5 |  |  | **40** | 55,6 | **102** | 41,1 |  |
| *Lauren classification* | | | | | | | | | | | |
| Intestinal | **49** | 68,1 | **94** | 47,74 | 0,003 |  | **9** | 16,4 | **137** | 62,8 | <0,001 |
| Diffuse | **11** | 15,3 | **30** | 15,2 |  |  | **26** | 47,3 | **15** | 6,9 |  |
| Mixed | **12** | 16,7 | **73** | 37,1 |  |  | **20** | 36,4 | **66** | 30,3 |  |
| Unclassified | 47 | | | |  |  | 47 | | | |  |
| *Growth pattern* | | | | | | | | | | | |
| Expansive | **26** | 31,3 | **30** | 13,6 | 0,001 |  | **8** | 11,4 | **49** | 20,7 | 0,114 |
| Infiltrative | **57** | 68,7 | **191** | 86,4 |  |  | **62** | 88,6 | **188** | 79,3 |  |
| Unclassified | 12 | | | |  |  | 13 | | | |  |
| *TNM stage* | | | | | | | | | | | |
| I/II | **53** | 60,9 | **128** | 55,9 | 0,48 |  | **36** | 50,0 | **149** | 60,1 | 0,14 |
| III/IV | **34** | 39,1 | **101** | 44,1 |  |  | **36** | 50,0 | **99** | 39,9 |  |
| *T stage* | | | | | | | | | | | |
| T1/T2 | **37** | 42,5 | **105** | 45,9 | 0,61 |  | **32** | 44,4 | **114** | 46,0 | 0,89 |
| T3/T4 | **50** | 57,5 | **124** | 54,1 |  |  | **40** | 55,6 | **134** | 54,0 |  |
| N stage | | | | | | | | | | | |
| N0 | **33** | 37,9 | **89** | 39,0 | 0,48 |  | **24** | 33,3 | **102** | 41,3 | 0,14 |
| N+ | **54** | 62,1 | **139** | 61,0 |  |  | **48** | 66,7 | **145** | 58,7 |  |
| Nx | 1 | | | |  |  | 1 | | | |  |
| *Resection margins* | | | | | | | | | | | |
| R0 | **78** | 90,7 | **202** | 88,2 | 0,34 |  | **59** | 81,9 | **225** | 91,1 | 0,028 |
| R1/R2 | **8** | 9,3 | **27** | 11,8 |  |  | **13** | 18,1 | **22** | 8,9 |  |
| No data | 1 | | | |  |  | 1 | | | |  |
| *Vascular invasion* | | | | | | | | | | | |
| Absent | **31** | 35,6 | **92** | 40,7 | 0,24 |  | **30** | 41,7 | **96** | 39,2 | 0,40 |
| Present | **56** | 64,4 | **134** | 59,3 |  |  | **42** | 58,3 | **149** | 60,8 |  |
| No data | 3 | | | |  |  | 3 | | | |  |
| *E-Cadherin* | | | | | | | | | | | |
| Loss | **19** | 21,8 | **52** | 22,6 | 1,00 |  |  |  |  |  |  |
| Retained | **68** | 78,2 | **178** | 77,4 |  |  |  |  |  |  |  |
| *S100P* | | | | | | | | | | | |
| Loss |  |  |  |  |  |  | **19** | 26,8 | **68** | 27,4 | 1,00 |
| Retained |  |  |  |  |  |  | **52** | 73,2 | **178** | 72,4 |  |

*Two cases did not have pathological data available.
